# Changing risk awareness and personal protection measures for low to high pathogenic avian influenza in live-poultry markets in Taiwan, 2007 to 2012

**DOI:** 10.1186/s12879-015-0987-8

**Published:** 2015-06-24

**Authors:** Ming-Der Liu, Ta-Chien Chan, Cho-Hua Wan, Hsiu-Ping Lin, Tsung-Hua Tung, Fu-Chang Hu, Chwan-Chuen King

**Affiliations:** College of General Education, Hungkuang University, Taichung (433), Taiwan; Center for General Education, National United University, Miaoli (360), Taiwan; Institute of Epidemiology and Preventive Medicine, College of Public Health, National Taiwan University, 17 Xu-Zhou Road, Taipei (100), Taiwan; Research Center for Humanities and Social Science, Academia Sinica, Taipei (115), Taiwan; Institute of Molecular and Comparative Pathobiology, School of Veterinary Medicine, National Taiwan University (NTU), Taipei (106), Taiwan; Institute of Clinical Medicine and School of Nursing, College of Medicine, National Taiwan University, Taipei (100), Taiwan

**Keywords:** Avian influenza, Cognition, Live-poultry market, Risk communication, Preventive measures, Public health policies, Taiwan

## Abstract

**Background:**

Outbreaks of low and high pathogenic avian influenza (LPAI, HPAI) H5N2 in chickens have occurred in Taiwan since 2003 and 2012, respectively. Fully understanding the different awareness, attitudes and protective behaviors adopted by workers in live-poultry markets (LPMWs) and local community residents (CRs) to face the challenges of LPAI and HPAI is very important to minimize viral adaptations to human populations.

**Methods:**

A structural questionnaire containing information on respondents’ occupation, personal risk awareness, attitudes toward different policies, and preventative measures was administered. The two-stage survey (before and after HPAI H5N2 outbreaks) was conducted from 2007 to 2012, including: (1) 430 LPMWs and 418 CRs at LPMs from different geographical areas of Taiwan after the government announced outbreaks of LPAI H5N2 during 2007–2009, and (2) 73 LPMWs and 152 CRs at two LPMs in central Taiwan after the HPAI H5N2 outbreaks in 2012. The chi-squared test and logistic regression were applied for univariate and multivariate analyses, respectively.

**Results:**

Before HPAI-H5N2 outbreaks, higher educated respondents demonstrated greater risk awareness and concerns regarding AI. However, LPM-workers protected themselves less from AI viruses (AIVs) and had lower acceptance of human or avian influenza vaccines. Most importantly, the participants who opposed (versus agreed with) the policy on banning live-poultry slaughtering at LPMs reported lower awareness of government prevention and control policies [Odds Ratio (OR): 0.76, 95 % Confidence Interval (CI): 0.56–1.01] or practiced preventive measures (OR: 0.42, 95 % CI: 0.25–0.70).

After HPAI-H5N2 outbreaks, the risk awareness about AI in central Taiwan significantly increased [LPAI to HPAI LPMWs: 34.6 to 65.6 %, *p* < 0.05; CRs: 44.0 to 76.5 %, *p* < 0.05] and LPMWs’ belief in the effectiveness of vaccination to prevent human or avian influenza virus infection strikingly decreased (92.3 to 68.5 %, *p* < 0.05).

**Conclusions:**

Risk awareness depends on high or low pathogenicity of AIVs, working in LPMs, levels of education, age, and proximity to the sites of severe AI outbreaks. Regardless of novel LPAI or HPAI virus reassortants that pose public health risks, prompt and clear risk communication focusing on both correct information about AIVs and the most appropriate preventive measures are important for effective prevention of human infection.

**Electronic supplementary material:**

The online version of this article (doi:10.1186/s12879-015-0987-8) contains supplementary material, which is available to authorized users.

## Background

Since the first occurrence of HPAI H5N1 human cases in Hong Kong in 1997, the public health threat of high pathogenic avian influenza (HPAI) has been a major global issue [1]. Exposure to live poultry was significantly associated with symptomatic or fatal cases of H5N1 [2]. As a result, Hong Kong government officials rapidly closed live-poultry markets (LPMs), and slaughtered more than 1.2 million chickens around the end of 1997 [3], thus successfully controlling the outbreak [4]. However, HPAI H5N1 viruses reappeared in 2003, spread across continents, and sickened 826 patients from 2003 to March 31, 2015 [5]. The overall case fatality rate was 53.3 % (404/826). Close contact with poultry is an important risk factor in H5N1 infection [6, 7].

In Southeast Asia, infections have mostly occurred in LPMs, where activities such as slaughtering, removal of feathers, customers touching chickens, transportation, and cleaning poultry waste occur very frequently [8, 9]. Importantly, most of the low pathogenic avian influenza (LPAI) H7N9 viruses, which caused human infections in different parts of China since February of 2013 [10], had high viral sequence identities to the H7N9 viruses isolated from wet poultry markets [11]. This was quite different from the avian influenza (AI) outbreaks in Europe and Africa, which occurred mostly in poultry farms where migratory birds played an important role [9]. Therefore, exposure to AI viruses (AIVs) in LPMs in Asia has been highly risky [12]. The increasing number of fatal cases due to H5N1 infections prompted the government of Hong Kong to initiate policies forbidding the slaughtering of live chickens or other poultry in wet markets [13].

The awareness of AI has been documented to affect a persons’ self-protection behaviors [14] and live poultry purchases [15]. It is important for mass media (such as television channels) to provide correct information to enhance the receivers’ knowledge and risk awareness [16]. An individual’s level of education [17], occupation (such as being poultry workers) [18, 19], and the residential area’s experiences with AI outbreaks [16, 20] may all affect a person’s perception of the risk of AI and their subsequent use of adequate personal protective equipment [21]. Thus, understanding all possible factors associated with risk awareness, attitudes, and practice of prevention measures (RAP), as well as differences in risk perception of outbreaks due to LPAI versus HPAI viruses between the live-poultry market workers (LPMWs) and community residents (CRs) are all important for providing further education and implementing public health policies on preventing AI infection.

Taiwan, with close proximity to these Asian AI epidemic and endemic centers, has many LPMs which could be potential sources of AI virus maintenance for emerging novel influenza reassortant viruses. The first AI outbreak of H5N2 in Taiwan started in December 2003, and subsequently these LPAI H5N2 viruses spread island-wide [22]. Although a policy to stamp them out was implemented from 2003 through 2004, this virus subtype remained in circulation for many years. In October 2008, another outbreak of H5N2 occurred in Kaohsiung (located in southern Taiwan), and a molecular analysis of the cleavage site of HA of the isolated virus indicated that it was high pathogenic. As the chicken pathogenicity index (intravenous pathogenicity index, IVPI) of the specimens collected for the second time was below 1.2 (IVPI = 0.89), the government officials announced and reported it as an outbreak of LPAI to the World Organization for Animal Health (OIE) (http://www.oie.int/) [23, 24]. In fact, both of these LPAI and HPAI H5N2 viruses are particularly unique, as they consist of reassortants of six internal segments derived from local Taiwan LPAI H6N1 viruses, but the HA and NA segments had the highest viral sequence identities with the 1994 Mexican-like H5N2 viruses [25, 26]. After the first HPAI H5N2 outbreak was officially announced on March 5, 2012, about 53,000 and 4,500 chickens were culled in Changhua and Tainan Counties in central Taiwan, respectively during February-March 2012 [27].

The elevation from LPAI H5N2 to HPAI H5N2 viruses in recent years in Taiwan provides a great opportunity to investigate whether the RAP of high-risk populations of those working in LPMs versus local CRs were different when facing the greater challenges of HPAI H5N2 viruses compared to the past LPAI H5N2 ones. Therefore, the specific aims of this study were: (1) to investigate the factors associated with high and low levels of RAP among LPMWs and CRs in outbreak areas throughout Taiwan immediately following the announcement by the government on outbreaks of LPAI H5N2; (2) to compare the differences in the factors associated with RAP after the outbreaks of these AI viruses with low versus high pathogenicity; and (3) to identify the different sources of information regarding the 2012 outbreaks of HPAI-H5N2 in chickens in central Taiwan among LPMWs and CRs as well as to compare their willingness to take preventive measures against LPAI-H5N2 and the other important emerging infectious diseases.

To the best of our knowledge, this is the first study to investigate public health awareness of both LPAI and HPAI of the same subtype viruses. Moreover, our findings could help public health administrators in areas or countries with LPAI to better prepare for possible subsequent HPAI outbreaks or minimize the numbers of human infections with either LPAI or HPAI viruses.

## Methods

### Study design, sampling strategy and study population

**Fig. 1:**
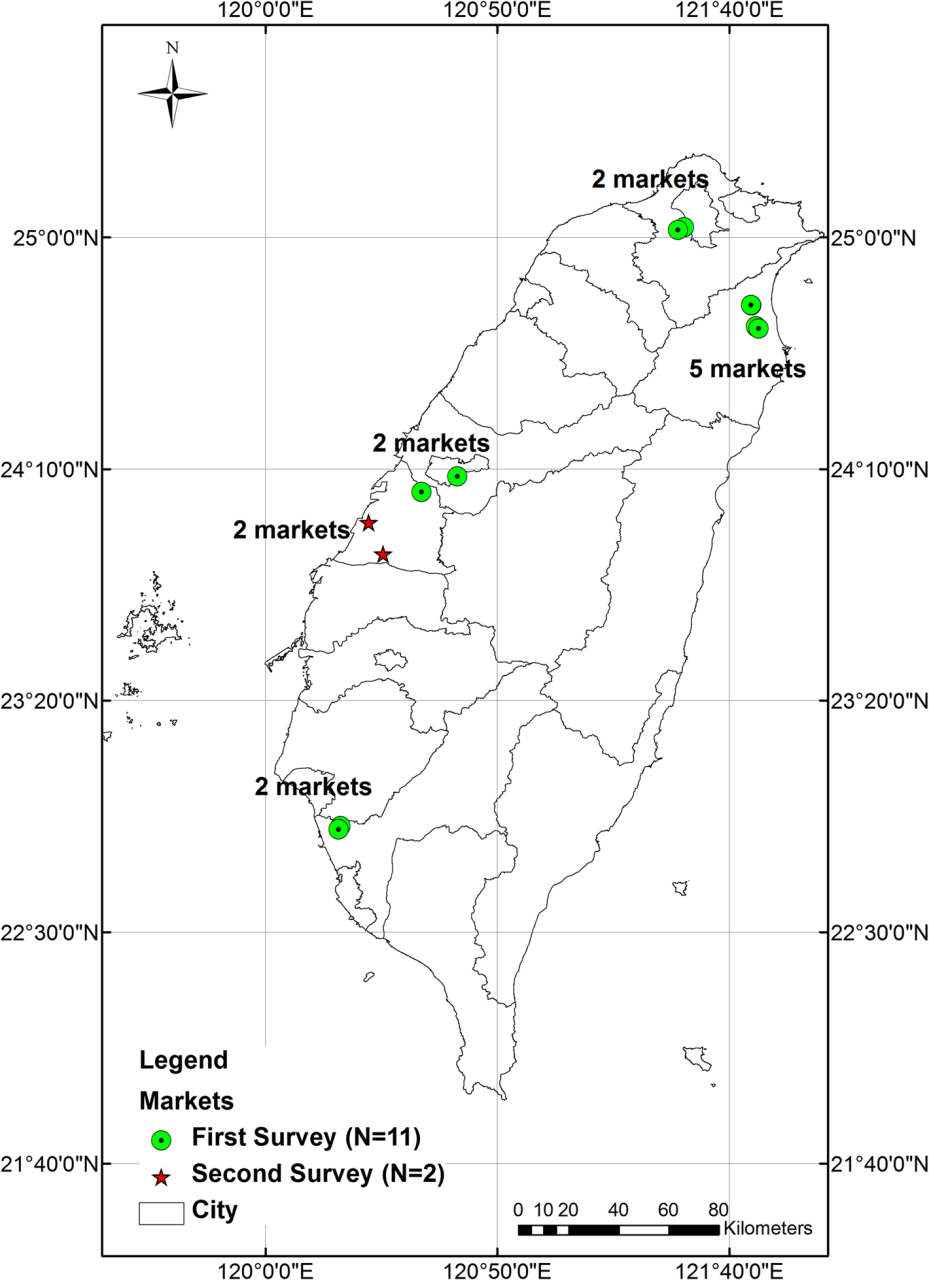
Geographical distributions of study sites (live-bird markets) in different parts of Taiwan. Location of markets selected for study during 1st survey in Taiwan from January 2007 to January 2009 (marked as circle in green), and markets during 2nd survey in Taiwan between February and March 2012 (marked as star in red)

To fully understand the differences in local responses after the outbreaks of LPAI and HPAI of H5N2 viruses in Taiwan, we conducted two-stage surveys including: (1) stage I: after the outbreaks caused by LAPI H5N2 viruses from January 2007 to January 2009, and (2) stage II: after the outbreaks caused by HPAI H5N2 viruses from February to March 2012. In the first-stage survey (before HPAI H5N2 outbreaks), 11 representative LPMs were selected across Taiwan, including two markets each in northern, central, and southern Taiwan, and five markets in eastern Taiwan as illustrated in Fig. 1. To increase the sample size for the areas with LPAI H5N2 outbreaks, which is the most neglected and important issue to be addressed, we covered all the major LPMs in the outbreak areas and asked as many people as possible to answer the questions at each study site. Since the outbreaks of LAPI H5N2 occurred in different years in various geographical areas, surveys were initiated in the wet markets in different time periods, right after the occurrence of outbreaks, including northern Taiwan (January to June 2007), central Taiwan (April to August 2008), eastern Taiwan (February to May 2008), and southern Taiwan (January 2009). After the outbreaks of HPAI H5N2, which were restricted to Changhwa County in central Taiwan between February and March 2012, the second-stage survey was conducted in the two LPMs situated in the outbreak county (shown in Fig. 1) with a smaller sample size from late June to early July 2012. For better comparison of the outcome measures between the two studied populations — (1) live-poultry market workers (LPMWs) as a “high-risk group” and (2) community residents (CRs) as a “low-risk group”, we used convenience sampling among these two groups for each of the study areas. The CRs, who were buyers but did not sell or touch any live poultry, were sampled from visitors who shopped at the same LPMs or visited the closest convenience stores (such as 7–11 stores) at the same time as we asked LPMWs.

### Questionnaire and data collection

A structural questionnaire was designed to investigate: (1) AI awareness, (2) knowledge of government policies, and (3) protection measures used. To achieve the study objective, the team members who designed and reviewed the questionnaire included infectious disease physicians, infectious disease epidemiologists, scholars experienced in knowledge, attitude and practice (KAP) of diseases, field workers who frequently went to LPM to take poultry specimens, and administrators in LPMs. The questionnaire included items such as demographic information, job duties, prevention measures, personal perceptions, the impact of China’s AI outbreaks on Taiwan, attitudes toward different policies such as killing poultry at LPMs, and potential confounding variables (age, gender, educational level, and living area) [Additional file 1: Appendix 1, Additional file 3: Appendix 3]. We did a pilot test on both study groups in different geographical areas to assure full understanding and reliability. After a comprehensive review by questionnaire design team members, the wording of the questionnaire was revised and simplified to maximize the response rates. There were five main questions measuring risk awareness, attitudes and personal protection measures (RAP) [Additional file 3: Appendix 3]. In addition to these five main questions, questions on the awareness of HPAI H5N2 outbreaks in 2012 and risk perception in LPAI-H5N2, HPAI-H5N2 and other important infectious diseases in Taiwan [such as severe acute respiratory syndrome (SARS)] were also included in the questionnaire of the second surveys for better comparison. Most questions were multiple choice, with a comprehensive range of choices or differential scales or rankings [Additional file 4: Appendix 4]. However, the second main question on possible future outbreaks of human cases of infection with AIVs in Taiwan was measured by the Likert scale. The questionnaire was administered by well-trained interviewers.

### Data analysis and statistical tests

For better assessment of the exposure levels, the LPM workers were further classified into three risk groups, based on their occupational exposures. The “high-risk group” included butchers and sellers of raw chicken or duck meat. The “moderate-risk group” covered sellers of cooked chicken or duck meet, beef, mutton, pork, and other raw meat sellers. The “low-risk group” included other workers. Regarding the risk levels among the market workers, the results showed that workers in all these three risk groups were located significantly more in northern Taiwan than in the rest of Taiwan (*p* = 0.01, Table 2). We then focused on the comparisons of all possible factors that may be associated with RAP; in particular, the differences on each question between LPMWs and CRs based on their occupation were analyzed. Only statistically significant differences between these two study groups are presented in Tables 3 and 5, with the covariate of “occupation” adjusted in multivariate analyses.

Demographic characteristics (including age, gender, living area and education level) were summarized as frequencies and percentages. In order to analyze the respondents’ answers, we classified their responses on RAP measures into a binary scale (positive and negative perception of the questions) and used a chi-square test and logistic regression for univariate and multivariate analyses, respectively. Additional file 4: Appendix 4 is the summary for all the assigned “positives” as “1 s” and “negatives” as “0 s” as a binary scale. For univariate analysis, a chi-squared test was used to compare differences in categorical variables [such as age: 17–40, 41–64 and≧65 (elderly)] and outcome of KAP measures between LPMWs and CRs. The outcome variables, explanatory variables, as well as the model performance for data analyses in this study are all summarized in Additional file 3: Appendix 3. The comparison of perception changes before and after chicken H5N2 outbreaks in central Taiwan was analyzed by two-proportion Z-test (Table 7).

For multivariate analysis, we pooled LPMWs and CRs together and then analyzed the outcome measures for each question. Then, logistic regression with stepwise selection of variables was used for estimating the adjusted odds ratios (OR) of explanatory factors and their 95 % confidence intervals (95 % CI) after adjusting for important confounding variables such as age [28], gender, residential area (northern, southern, central and eastern Taiwan), education level, and occupation (i.e., LPM-workers and community residents), plus the other outcome variables in addition to the asked RAP questions for both LPAI and HPAI surveys in Tables 3 and 5, respectively. For example, the last question, on effectiveness of vaccines, and the variables from all other questions in the same RAP Table such as impact of China on Taiwan were entered for better assessment. For the best statistical performance, both age and education were entered as continuous variables. Only significant variables (*p* < 0.05) were included in our final model. Therefore, we controlled for the regional differences in each analyses of both the 1st- and 2nd-stage surveys.

To ensure the validity of our results, basic model-fitting techniques for (1) variable selection, (2) goodness-of-fit (GOF) assessment, and (3) regression diagnostics were all used in our regression analyses. The statistical analysis was performed using SAS 9.1.3 (SAS Institute, Cary, NC, U.S.A.). Variables with *p*-value less than 0.05 were considered statistically significant. Cox & Snell R-square and Nagelkerke R-square were applied and the results listed in Additional file 3: Appendix 3.

### Ethics

The study and its consent procedures were approved by the Ethical Committee of National Taiwan University Hospital (Approval Number: 201101069RC). Respondents were informed of the purpose of the study, while oral consent was obtained before anonymous questionnaires were administered. Due to concern for privacy of the Chinese signatures of names, written informed consent was not collected. Whenever the respondents did not agree to join the study, the interviewers respected their opinions and did not continue for those cases. For the respondents aged less than 18 years, the interviewers first got the agreement of their parents or guardians. Otherwise, the interviewers dropped these cases. In other words, all the successfully collected questionnaires were agreed to verbally by the adult respondents themselves or children’s parents or guardians. In addition, our data were fully de-identified to protect the respondents’ privacy, and only group data were used for further analyses and statistical tests.

## Results

### Demographic analyses and response rates of the study populations

Both surveys recruited LPMWs and CRs. The response rates for high-, moderate-, and low-risk groups of LPMWs and CRs in the stage I survey were 98 %, 95 %, 93 %, and 90 %, respectively. Such rates for LPMWs and CRs in the stage II survey were 80 % and 90 %, respectively. The response rates for LPM workers after the HPAI H5N2 outbreaks were lower than those after the LPAI H5N2 outbreaks.

In the first-stage survey after the LPAI H5N2 outbreaks but before the HPAI H5N2 outbreaks, a total of 848 questionnaires were administered, including 430 to LPMWs and 418 to CRs (Table 1). In stage I, there were significant differences in gender and education, but the results were comparable across age and geographical distributions, without statistical differences between these two groups [Tables 1 and 2]. However, workers in the wet markets had a significantly higher proportion of males [52.6 % (226/430)] as compared to the CRs [29.9 % (125/418)] (*p* < 0.001). Overall, the CRs had higher levels of education than the LPMWs (*p* < 0.001).

**Table 1:** Distribution of variables among two study populations during the study period after LPAI H5N2 outbreaks (Stage I) in Taiwan, 2007–2009

Characteristics		Two study populations				*p*-value
		Live-poultry market workers		Community residents		
		*N* = 430	%	*N* = 418	%	
Age (Years)	17-40	141	32.8	165	39.5	0.06
	41-64	268	62.3	241	57.7	
	≥65	21	4.9	12	2.9	
Gender	Male	226	52.6	125	29.9	<0.001*
	Female	204	47.4	293	70.1	
Geographical Areas	North	195	45.3	193	46.2	0.97
	Central	104	24.2	100	23.9	
	South	61	14.2	55	13.2	
	East	70	16.3	70	16.7	
Education	≦Elementary	89	20.7	50	12.0	<0.001*
	Junior high	133	30.9	95	22.8	
	Senior high	169	39.3	157	37.6	
	≧College	39	9.1	115	27.6

**Table 2:** Distribution of demographic variables among live-poultry market workers by levels of risk in the study period after LPAI H5N2 outbreaks (Stage I) in Taiwan, 2007–2009

		High risk§		Moderate risk§		Low risk§		
Variables		*N* = 73	%	*N* = 113	%	*N* = 244	%	*p*-value
Age (Years)	17–40	24	32.9	34	30.1	83	32.8	0.95
	41–64	45	61.6	74	65.5	149	62.3	
	≧65	4	5.5	5	4.4	12	4.9	
Gender	Male	44	60.3	65	57.5	117	48.0	0.09
	Female	29	39.7	48	42.5	127	52.0	
Geographical Areas	North	33	45.2	40	35.4	122	50.0	0.01*
	Central	16	21.9	38	33.6	50	20.5	
	South	8	11.0	11	9.7	42	17.2	
	East	16	21.9	24	21.2	30	12.3	
Education	≦Elementary	22	30.1	20	17.7	47	19.3	0.27
	Junior high	16	21.9	40	35.4	77	31.6	
	Senior high	30	41.1	44	38.9	95	38.9	
	≧College	5	6.8	9	8.0	25	10.2	

In Stage I, the mean, median, and range of age for CRs were 43.6 ± 11.4, 44.0, and 18–84, respectively whereas those for live poultry market workers (LPMWs) were 45.8 ± 11.3, 47.0, and 17–87, respectively. We used a chi-square test for the statistical analyses in Table 1. There was no significant difference between these two groups [Table 1]

The data within the percentages of community residents related to the different demographical variables in the Stage I survey served as the reference group in this Tables 1 and 2

§High Risk: butcher, raw chicken/duck sellers. Moderate Risk: Sellers of cooked chicken/duck, beef, pork, mutton, and/or other raw meat. Low Risk: Market cleaners, administrative officers, and those selling flowers, dry goods, vegetables and fruits. *p-value < 0.05

In the second stage survey (after the outbreaks of HPAI H5N2 in central Taiwan), 225 respondents (73 LPMWs and 152 CRs) completed the questionnaires. In this subgroup (Table 4), the LPMWs were significantly less educated (*p* < 0.001) and older [mean ± standard deviation (S.D.) of age (by years): 49.1 ± 14.6 vs. 32.2 ± 13.5, *p* < 0.001)] than those CRs of the same local areas with HPAI outbreaks.

### Factors associated with risk awareness, attitude and preventive measures of AI before the outbreaks of HPAI H5N2

Firstly, we analyzed possible factors influencing the awareness of AI in stage I before the outbreaks of HPAI H5N2 in Taiwan (Table 3). As to the impact of China on Taiwan (Question 1 of Table 3), the respondents with higher levels of education thought that the external outbreaks of AI in poultry or in human cases in China would affect Taiwan (OR: 2.09, 95 % CI: 1.48–2.95), whereas those who opposed the ban on live poultry slaughter in Taiwan’s traditional markets did not believe in such an influence (OR: 0.34, 95 % CI: 0.18–0.64).

**Table 3:** Risk awareness, attitudes, and protection behaviors against avian influenza in period after LPAI H5N2 outbreaks (Stage I) in Taiwan, 2007–2009

5 Surveyed questions	Variables	ORs	95 % CI
1. Taiwan will be affected by the outbreaks of influenza in China	Educational Status	2.09	1.48–2.95
	Oppose ban on live poultry slaughtering in traditional markets	0.34	0.18–0.64
2. Taiwan residents will become infected with avian influenza (AI)	Educational Status	1.42	1.19–1.69
	Believe outbreak of AI in China will affect Taiwan	2.22	1.55–3.18
	Support ban on live poultry markets	1.51	1.10–2.06
	Southern Taiwan^a^	3.27	2.01–5.31
	Central Taiwan^a^	0.59	0.41–0.84
	Age^b^	0.98	0.97–1.00
3. Knowing new “Ten No’s, Five Needs” policy	Educational Status	1.26	1.08–1.46
	Central Taiwan^a^	3.37	2.42–4.70
	Eastern Taiwan^a^	3.87	2.63–5.71
	Believe Taiwan residents will not be infected with avian influenza	0.58	0.36–0.91
	Oppose ban on live poultry slaughter in traditional markets	0.76	0.56–1.01
4. Willing to take self-protection measures against avian influenza viral infection	Live-poultry market workers^c^	0.47	0.28–0.80
	Believe AI cases will appear in Taiwan	2.28	1.13–4.60
	Aware of new “10 No’s, 5 Needs” policy	2.41	1.46–3.97
	Eastern Taiwan^a^	0.53	0.28–1.00
	Believe outbreaks of AI from Mainland China will not affect Taiwan	0.14	0.07–0.28
	Have no opinions on banning birds from being slaughtered in traditional markets	0.42	0.25–0.70
5. The vaccine will provide effective protection against avian influenza viral infection	Live-poultry market workers^c^	0.30	0.17–0.50
	Believe AI from Mainland China will not affect Taiwan	0.21	0.09–0.46
	Aware of “Ten No’s, Five Needs” policy	0.52	0.31–0.88
	Believe it is unnecessary to protect oneself against AI viral infection	0.19	0.10–0.35
	Central Taiwan^a^	0.10	0.01–0.83
	Eastern Taiwan^a^	0.05	0.01–0.39
	Northern Taiwan^a^	0.03	0.00–0.24

We used logistic regression for the statistical analyses in Table 3. For better statistical performance, education status was only significant as a “continuous variable” in Question #1 to #3 but not as a “dummy categorical variable”

*CI* Confidence Interval

^a^Other areas as a reference

^b^Age: A continuous variable

^c^Community residents as the control group

* p<0.05

** p<0.01

*** p<0.001

Next, the risk awareness regarding the impact of domestic AI outbreaks was assessed (Question 2 of Table 3). Besides level of education, respondents’ age and residential area also influenced their risk awareness of human infection of AI in Taiwan after the local LPAI outbreaks. The older participants (OR, 0.98; 95 % CI, 0.97–1.00) and those living in central Taiwan (OR, 0.59; 95 % CI, 0.41–0.84), where the population density of chickens is the highest and outbreaks of LPAI-H5N2 frequently occurred, were less likely to think that Taiwan residents would get infection with AIVs. However, the respondents living in the H5N2 epidemic site of Kaohsiung County in southern Taiwan in January 2009 with different awareness of AI (after the controversial judgment on the causing agents as LPAI or HPAI viruses in the 2008 outbreak) compared with those in other areas, perceived that people in Taiwan would become more likely to be infected with AIVs (OR: 3.27, 95 % CI: 2.01–5.31).

Since the government announced the new policy of “Ten No’s, Five Needs” in 2005 (Additional file 2: Appendix 2) after many outbreaks of LPAI H5N2, we then investigated the factors associated with knowing this policy and a possible future ban on slaughtering live poultry in traditional markets (Question 3 of Table 3). The results showed that greater percentages of respondents with higher levels of education or living in central or eastern Taiwan knew the contents of the new government policy on AI than those in other areas (central Taiwan OR, 3.37; 95 % CI, 2.42–4.70 and eastern Taiwan OR, 3.87; 95 % CI, 2.63–5.71). On the other hand, those who had less belief that human cases of AI would not happen in Taiwan (OR: 0.58, 95 % CI: 0.36–0.91) and those who were less opposed to banning live poultry slaughtering in LPMs (OR: 0.76, 95 % CI: 0.56–1.01) had knowledge of this new policy.

Most importantly, we evaluated self-protection measures that are very crucially important to preventing AI infection (Question 4 of Table 3). LPMWs (compared to CRs: OR, 0.47; 95 % CI, 0.28–0.80), the respondents from eastern Taiwan, where fewer outbreaks were reported (OR, 0.53; 95 % CI, 0.28–1.00), and those without opinions on the banning of poultry slaughtering (OR, 0.42; 95 % CI, 0.25–0.70), were all less motivated than those in the comparison groups to implement self-protection measures against AIVs. By contrast, both respondents who believed human cases of AI would appear in Taiwan (OR: 2.28, 95 % CI: 1.13–4.60) and those who were also more aware about the new policy“Ten No’s, Five Needs” (OR: 2.41, 95 % CI: 1.46–3.97) had more willingness to engage in self-protection against AIVs. In other words, the study subjects’ awareness of risk on AI in Taiwan and attitude in supporting or opposing government policies were associated with their taking subsequent personal prevention and control measures. On the contrary, those who believed that AI outbreaks from mainland China would not have an impact on Taiwan did not implement self-protection measures against AIVs (OR, 0.14; 95 % CI, 0.07–0.28).

Regarding the perceptions on effectiveness of influenza vaccines (Question 5 of Table 3), market workers were more likely to have a negative perception about the effectiveness of avian or human influenza vaccines to protect against human infection with AIVs (OR: 0.30, 95 % CI: 0.17–0.50). Similarly, the respondents living in northern Taiwan (OR: 0.03, 95 % CI: 0.00–0.24), central Taiwan (OR: 0.10, 95 % CI: 0.01–0.83) and eastern Taiwan (OR: 0.05, 95 % CI: 0.01–0.39), compared to those from other areas also had lower perceptions of the effectiveness of any influenza vaccine. All the odds ratios for flu vaccines were less than one during the period before official announcement of the HPAI H5N2 outbreaks.

### Factors associated with risk awareness, attitude and preventive measures after the outbreaks of HPAI H5N2

The results of the risk awareness, attitudes about, and protective behaviors against AI after the outbreak of HPAI H5N2 in Taiwan are summarized in Table 5. The older respondents were less likely to believe that Taiwan would be affected by the influenza outbreaks in mainland China (OR: 0.85, 95 % CI: 0.78–0.93, Question 1). Again, the impact of outbreaks of AI abroad as well as in Taiwan was further explored (Questions 1 and 2). Interestingly, there was a strong association between the respondents’ beliefs about Taiwan’s human outbreaks of AIV cases and influenza outbreaks in China (OR: 25.51, 95 % CI: 4.24–153.66). Moreover, the respondents who perceived that the outbreaks in China would lead to human infection of AIV in Taiwan and those who believed that Taiwan would face an AI threat reported that they had more willingness to take preventive measures against AIVs (OR, 6.83; 95 % CI, 2.10–22.26; OR, 3.88; 95 % CI, 1.16–12.98, respectively). Besides the impact of mainland China, the participants who were aware of the critical condition of the one H5N1 pediatric case in Hong Kong in June 2012 were more likely to have good knowledge of the new “Ten No’s, Five Needs” policy (Question 3, OR, 4.24; 95 % CI, 2.09–8.59).

For public health prevention, both personal protection and vaccination (Questions 4 and 5 of Table 5) were examined. The Taiwanese respondents knowing of the critical condition of the 2012 Hong Kong H5N1 pediatric case (OR: 5.85, 95 % CI: 1.45–23.56), or believing in the possibility of Taiwan’s domestic risk of future human infection of AIVs (OR: 4.09, 95 % CI: 1.15–14.62), or having knowledge of the severity of the threat posed by the AI viruses (OR: 6.62, 95 % CI: 1.54–28.55) all reported the habit of carrying out self-protection measures for preventing AI viral infection. For other preventive measures, only participants who believed in the effectiveness of seasonal influenza vaccines in protecting against either human or avian flu (OR: 5.51, 95 % CI: 1.97–15.42) or both types of flu (OR: 7.65, 95 % CI: 2.61–22.43) would consider receiving AI vaccination. Noteworthily, LPMWs were less likely to accept AI vaccine than those CRs in central Taiwan with HPAI outbreaks [61.6 % (45/73) vs. 75.0 % (114/152), *p* = 0.04] (Table 4).

**Table 4:** Distributions of demographical variables and avian influenza vaccine acceptability among respondents (interviewed in poultry markets or community residents) from Changhwa County in Central Taiwan after Chicken HPAI H5N2 outbreaks (Stage II Survey)

Variables		Two study populations				*p*-value
		Live-poultry market workers		Community residents		
		*N* = 73	%	*N* = 152	%	
Age (Years)	11–40	18	24.7	106	69.7	<0.001*
	41–64	46	63.0	41	27.0	
	≧65	9	12.3	1	3.3	
	Missing	0		4		
Gender	Male	28	38.4	48	36.4	0.78
	Female	45	61.6	84	63.6	
	Missing	0		20		
Education	≦Elementary	21	28.8	2	1.4	<0.001*
	Junior high	16	21.9	11	7.7	
	Senior high	22	30.1	44	31.0	
	≧College	14	19.2	85	59.9	
	Missing	0		10		
Acceptance of avian influenza vaccine^a^	Yes	45	61.6	114	75.0	0.04*

In Stage II, the mean, median, and range of age for CRs were 32.2 ± 13.5, 30.0, and 13–73, respectively whereas those for LPMWs were 49.1 ± 14.6, 50.0, and 11–87, respectively. We used a chi-square test for the statistical analyses in Table 4. LPMWs were significantly older than CRs (*p* < 0.001)

*p-value < 0.05. The data within the percentages of community residents related to the different demographical variables in the Stage II survey served as the reference group in this Table 4

^a^Our government officials initiated the pilot study of phase 1 H5N1 avian influenza vaccine trial for animal-related workers in 2009. At that time, the acceptance rate was quite low. Therefore, the data of the reported “acceptance of avian influenza vaccine between live-poultry market workers and community residents” were thus compared only after the 2nd survey in Table 4

The risk awareness of AI causing serious disease and even death was evaluated (Question 6 of Table 5). CRs of areas with documented chicken HPAI H5N2 outbreaks had higher awareness of AI leading to severe human clinical cases or fatalities (OR: 3.64, 95 % CI: 1.03–12.86) than CRs of other areas. These respondents with greater alertness of the AI severity not only had better knowledge of the new “Ten No’s, Five Needs” policy (OR, 4.10; 95 % CI, 1.19–14.12) but also were more likely to take preventive measures against AIVs (OR, 4.38; 95 % CI, 1.08–17.76).

**Table 5:** Risk awareness, attitude and protection behaviors against avian influenza after chicken HPAI H5N2 outbreaks (Stage II survey) in Taiwan

Questions		ORs	95 % CI
Variables			
1. Taiwan will be affected by the outbreaks of influenza in China	Believe Taiwan residents will become infected with AIVs	25.51	4.24–153.66
	Age	0.85	0.78–0.93
2. Taiwan residents will become infected with avian influenza (AI)	Believe influenza outbreaks in China will affect Taiwan	6.83	2.10–22.26
	Will take preventive measures against AI	3.88	1.16–12.98
3. Knowing new “Ten No’s, Five Needs” policy^a^	Aware of the critical condition of the child in Hong Kong infected with H5N1	4.24	2.09–8.59
4. Willing to take self-protection measures against avian influenza viral infection	Aware of critical condition of the child in Hong Kong infected with H5N1	5.85	1.45–23.56
	Believe people in Taiwan will be infected with AIVs	4.09	1.15–14.62
	Know AI may cause serious diseases and death	6.62	1.54–28.55
5. Willing to receive avian influenza vaccination	Believe seasonal flu vaccines can reduce chance of getting human flu or AI	5.51	1.97–15.42
	Believe seasonal flu vaccines can reduce chance of getting human flu and AI	7.65	2.61–22.43
6. Know AI may cause serious illness and even death	Know the “Ten No’s, Five Needs” policy	4.10	1.19–14.12
	Community Residents^b^	3.64	1.03–12.86
	Will take preventive measures against AI	4.38	1.08–17.76

We used logistic regression for the statistical analyses in this Table 5

*Age* continuous variable, *AIV* Avian influenza viruses

*CI* Confidence Interva

^a^Ten No’s, Five Needs policy in Appendix 2

^b^Live-poultry market workers as the control group

After the government declared the outbreaks of HPAI H5N2 in Taiwan in 2012, we found protective behaviors and shopping habits were different between LPMWs and CRs. Among the 221 respondents, 81 % of them washed their hands frequently (179/221) and 75.1 % of them (166/221, with 4 missing values) reported the intention to wear facemasks to protect themselves once AI outbreaks occur (Table 6). In this study, we did not differentiate surgical masks from cloth masks in our questionnaire on “facemasks”. However, most of the public can easily buy surgical masks in convenience stores or drug stores. Even among the CRs, high percentages of them intended to change their shopping behaviors such as avoiding both live-poultry markets (74/152, 48.7 %) and poultry purchases (56/152, 36.8 %).

**Table 6:** Protection measures adopted by respondents in Central Taiwan after the HAPI H5N2 outbreak in 2012

Protection measures^a^	Two study populations		*p*-value
	Live-poultry market workers	Community residents	
	(*n* = 69^b^)	(*n* = 152)	
Wash hands frequently	58(84.1 %)	121(79.6 %)	0.43
Wear facemasks	50(72.5 %)	116(76.3 %)	0.54
Comply with government’s policy	27(39.1 %)	89(58.6 %)	<0.01**
Do Exercise	14(20.3 %)	66(43.4 %)	<0.01**
Obtain more information	6(8.7 %)	61(40.1 %)	<0.001***
Receive human flu vaccine	17(24.6 %)	49(32.2 %)	0.25
Receive AI H5N1 vaccine	8(11.6 %)	53(34.9 %)	<0.01**
Take Tamiflu	3(4.3 %)	12(7.9 %)	0.33
Take Chinese herbs	6(8.7 %)	6(3.9 %)	0.15
Stop going to LPMs^c^	-	74(48.7 %)	-
Stop buying poultry in LPMs^c^	-	56(36.8 %)	-

We used chi-square test for the statistical analyses in Table 6

The data within the percentages of community residents related to the different preventive measures served as the reference group in this Table

*LPMs* Live-poultry markets

^a^The answers are multiple choices

^b^Total N = 73, with four missing values

^c^Only asked for community residents in Central Taiwan

### Differences in the factors associated with RAP after the outbreaks of these AI viruses with regards to low versus high pathogenicity

Comparing the perception differences before and after the outbreaks of chicken HPAI H5N2 among the study participants only in central Taiwan, our results revealed significant increases in the proportion of both LPMWs and CRs who perceived Taiwanese will be infected by AIVs (Table 7). After the occurrence of domestic HPAI H5N2 outbreaks, the LPMWs’ risk perception on the possibility of AI epidemics in mainland China affecting Taiwan significantly decreased (94.2 to 69.9 %, p < 0.05), but their risk awareness on the likelihood of people in central Taiwan being infected with AIVs strikingly increased (from 34.6 to 65.6 %, *p* < 0.05). However, the LPMWs’ belief that vaccines are capable of preventing human or avian influenza virus infection strikingly decreased (92.3 to 68.5 %, *p* < 0.05).

**Table 7:** Changes in perception among study participants before and after the 2012 chicken HPAI H5N2 outbreaks in Central Taiwan

Perception changes	Live-poultry market workers					Community residents				
	Before HPAI		After HPAI		*p*	Before HPAI		After HPAI		*p*
	*N*	%	*N*	%		*N*	%	*N*	%	
1. AI epidemics in China will affect Taiwan	104	94.20 %	73	69.9 %	<0.001*	100	94.00 %	150	99.3 %	0.013*
2. People in Taiwan will be infected by AIVs	104	34.60 %	64	65.6 %	<0.001*	100	44.00 %	149	76.5 %	<0.001*
3. Respondents knew government (Ten No’s, Five Needs) policy	104	58.70 %	70	68.60 %	0.186	100	66.00 %	147	68.70 %	0.656
4. Respondents will take self-protection measures against AIVs	104	91.30 %	72	81.90 %	0.064	100	95.00 %	152	96.70 %	0.499
5. Vaccination can prevent human or avian influenza virus infection	104	92.30 %	73	68.5 %	<0.001*	100	95.00 %	152	94.70 %	0.916

This survey was implemented during late June-July 2012, after the outbreak of HPAI H5N2

Data in the two columns of “Before HPAI for live-poultry market workers” (LPMWs) and “Before HPAI for community residents” (CRs) served as two reference groups of LPMWs and CRs, respectively

*N* Number of participants who answered that specific question

*p-value < 0.05 *, using a two-proportion Z-test

### Sources of information regarding the 2012 outbreaks of HPAI in chickens in central Taiwan among live poultry market workers versus community residents

After the incident of HPAI-H5N2 outbreaks in central Taiwan, we asked participants whether they knew that HPAI outbreaks had occurred there. Among those who knew about the HPAI H5N2 outbreaks, LPMWs had significantly paid more attention to the 2012 AI outbreaks than CRs [87.7 % (64/73) vs. 78.3 % (119/152), *p* = 0.03] (Table 8). Detailed analysis of the sources of information on these HPAI outbreaks (Table 5) showed that the major channel for receiving information on the outbreaks for both groups was television broadcasts (LPMWs vs CR: 90.6 % vs 84.9 %, *p* = 0.27), followed by the internet and relatives or friends for CRs (37 %) and newspapers for LPMWs (15.6 %). However, seeking information through newspapers, internet and radio broadcasts was statistically more common among the CRs than the LPMWs (newspapers: 34.5 % vs. 15.6 %, *p* < 0.01; internet: 37.0 % vs. 10.9 %, *p* < 0.001; radio: 12.6 % vs. 1.6 %, *p* < 0.05).

**Table 8:** Sources and channels of information about 2012 outbreaks of highly pathogenic avian influenza in chickens in Central Taiwan (Stage II Survey)

Aware of HPAI	Two study populations				*p*-value
	Live-poultry market		Community		
	Workers (*n* = 64)		Residents (*n* = 119)		0.03^a,b^
Sources	n	%	n	%	
Television	58	90.6	101	84.9	0.28
Newspapers	10	15.6	41	34.5	<0.01
Internet	7	10.9	44	37.0	<0.001
Relatives or Friends	5	7.8	18	15.1	0.16
Radio	1	1.6	15	12.6	<0.05
Other Market Workers	4	6.3	3	2.5	0.21
Phone Calls/Messages	0	0.0	1	0.8	0.48

The data within the percentages of community residents related to the sources or channels of information served as the reference group in this Table 8

*P*-values: Chi-square test was used for the statistical analyses

^a^There were 73 live-poultry market workers, 64 of whom (87.7 %) knew about the HPAI outbreaks in central Taiwan

^b^There were 152 live-poultry market workers, 119 of whom (78.3 %) knew about the HPAI outbreaks in central Taiwan

### Risk perceptions of LPAI H5N2, HPAI H5N2 and other important emerging infectious diseases

Comparing the respondents’ risk perception of LPAI H5N2, HPAI H5N2 and other important emerging infectious diseases (EIDs) versus the old disease of tuberculosis (Table 9), severe acute respiratory syndrome (SARS) was perceived as the most risky infectious disease by the respondents, while HPAI H5N2 was thought much more important than LPAI H5N2, particularly among market workers (HPAI vs LPAI for LPMWs: 64.4 % vs 31.5 %, *p* <0.05; for CRs: 54.6 % vs 33.6 %, *p* <0.05).

**Table 9:** Willingness to take preventive measures for the selected infectious diseases (Stage II Survey)

Infectious diseases^a^	Two study populations		*p*-value
	Live-poultry market workers (*N* = 73)	Community residents (*N* = 152)	
Severe Acute Respiratory Syndrome (SARS)	60 (82.2 %)	112 (73.7 %)	0.159
HPAI H5N2	47 (64.4 %)	83 (54.6 %)	0.164
2009 Pandemic H1N1	46 (63.0 %)	101 (66.4 %)	0.612
Enterovirus	24 (32.9 %)	86 (56.6 %)	0.001*
LPAI H5N2	23 (31.5 %)	51 (33.6 %)	0.760
Tuberculosis	21 (28.8 %)	76 (50 %)	0.003*

The data within the percentages of community residents related to the willingness to take preventive measures for the selected infectious diseases served as the reference group in this Table 9

*HPAI* Highly pathogenic avian influenza

*LPAI* Lowly pathogenic avian influenza

*p-value <0.05 by chi-square test

^a^Numbers in this table indicate how many respondents indicated they were willing to take preventive measures against each disease listed. Respondents were given a list and were free to select any or all of the infectious diseases (that they would protect themselves from)

## Discussion

Global epidemiology of AI has focused mostly on human cases after the outbreaks of HPAI [29, 30], with little attention to LPAI. To our knowledge, this is the first study to compare the differences in risk awareness, attitude and personal protection practice (RAP) right after the outbreaks of both LPAI and HPAI of the same virus subtype. We have the following five major findings that may help future global efforts to prevent novel AI viruses (AIVs) with pandemic threat to human populations. First, risk awareness, positive attitudes and taking preventive measures depend on several factors, including high or low pathogenicity of AIVs (HPAIVs or LPAIVs), working in LPMs, level of education, age, proximity to the sites of severe AI outbreaks, knowledge of AI outbreaks in neighboring countries or areas (e.g., mainland China or Hong Kong), the level of understanding of important knowledge on AIVs, and learning preventive measures through various channels of mass media. Second, respondents with higher risk perception (concerning human AI infections in Taiwan) before HPAI outbreaks had not only more awareness about the AI outbreaks in mainland China affecting Taiwan, but also better attitudes toward meeting domestic needs (endorsing the government’s new policy on AI, and supporting a ban on slaughtering live poultry in markets). Third, participants’ better attitudes towards AI prevention and control were associated with higher motivation to practice self-protection measures, even in preventing LPAIVs. Fourth, individuals with lower educational levels, the LPM workers with high exposure to AIVs, and the respondents living in areas with low frequency of AI outbreaks had a lower risk awareness of AIVs, particularly LPAIVs that might be transmitted to humans. Fifth, the respondents’ risk awareness and protective behaviors during the periods of LPAI H5N2 outbreaks strikingly rose after experiencing the outbreaks of HPAI H5N2. All these together suggest that neglecting health education and precautions in LPMs might facilitate adaptation of the virus in human populations, particularly the silent spreading of LPAIVs.

### High-risk populations in live-poultry markets

Among all the factors associated with RAP related to human infection of AIVs, the pathogenicity of AI virus is crucially important, particularly in those areas or countries with no prior experience of HPAI outbreaks. However, most past studies have targeted poultry workers as the high risk population due to exposure to possible HPAIVs of H5 and H7 in sick or dead poultry [31], neglecting the dynamic changes of AIVs from LPAIVs to HPAIVs. Our study showed that risk perceptions changed significantly for both market workers and the general population after HPAI outbreaks in Taiwan. The increased pathogenicity of H5N2 AIVs may have caused the study subjects to feel nervous, as they faced the outbreaks of SARS in 2003 and the novel H1N1 influenza pandemic in 2009, thus raising risk perceptions. Lower risk perception in these high-risk populations is a general problem in different parts of the world, including Taiwan [19], Italy, Thailand and China [18]. This shows that high-exposure workers need more appropriate information on AIVs to complement the information through mass media, which is usually obtained after rather than before outbreaks. Generally, LPMWs before the outbreaks of HPAI in this study also had a lower perception of Taiwanese AI risk than local residents (Question 2 in Table 7), so they did not adopt any preventive measures to avoid AIV infection, and did not believe the seasonal influenza vaccination was effective for preventing human or avian influenza.

Live-poultry markets, the major interface areas between poultry and humans offering conditions for sustainability, amplification, reassortment and cross-species transmission of AIVs, from H5N1 HPAIVs in 1997 [6] to the most recent H7N9 LPAIVs in 2013–2015, have been involved in many human-acquired AIV infections [32]. Most importantly, chickens sold in traditional LPMs can transmit AIVs to humans through respiratory transmission [33]. Interestingly, the older participants, who had more traditional thinking, and those living in central Taiwan, where the density of layer-chickens ranks the highest and outbreaks of LPAI-H5N2 occurred more frequently than other areas, had lower risk awareness of AIVs. By contrast, the respondents who lived in the epidemic site of Kaohsiung County in southern Taiwan, where the cleavage site of hemagglutinin (HA) was identified as HPAIVs in 2008, had higher perceptions than residents in other areas (OR: 3.27) that people in Taiwan would become infected with AIVs.

After the outbreaks of chicken HPAI H5N2, the LPMWs still had a lower belief in the effectiveness of vaccination to prevent human or avian influenza virus infection, regardless of their job duties. Furthermore, compliance with and understanding of the government policy raised the individual’s risk perception from LPAIVs to HPAIVs, while other measures of risk awareness had fewer differences among these two surveys, except for the reduction in risk perception on possible AI outbreaks in mainland China affecting Taiwan among LPMWs in central Taiwan. Such a striking decrease can be explained by the occurrence of the local HPAI outbreaks instead of the imported infections. In other words, attitudes became positive and preventive measures were reported to be taken when they faced the threat of HPAIVs. This may have been influenced by mass media or the experience of getting a voluntary H5N1 AI vaccine. Therefore, our study demonstrated that the perception of AI risk was elevated as the pathogenicity of AIVs changed from low to high. These results emphasize the public health significance of educating high-risk populations, starting from LPAIVs with capability to donate viral gene segments for generating novel reassortant viruses that may increase infectivity to humans, like H7N9 and H10N8 in China [34, 35] and H5N2 [25, 26] and H6N1 [36] in Taiwan.

### Public health measures and policies

Three important policies for AI prevention and control measures are closure of LPMs, practicing personal protective measures (PPM), and receiving influenza vaccines before flu seasons. In general, numerous LPMs are widely distributed in urban areas that might facilitate avian-to-human and subsequent human-to-human transmissions of novel influenza viruses, unlike poultry farms, which are more frequently located in rural areas. Such geographical differences between the urban and rural areas affected the awareness of AI in Turkey [37] and in China [38]. In the past, closure of LPMs has been implemented after the confirmation of severe or fatal human cases of H5N1 and H7N9 [39]. However, the virus may still reemerge after temporary closure. Therefore, market shutdown is not the most effective long-term measure, especially as the stake-holders are not likely to support it. The most likely approaches are: (1) weekly and monthly off-market days (such as Mondays and Chinese festivals in Taiwan) for cleaning and interrupting viral transmission, (2) banning the slaughter of live poultry, (3) practicing PPM, and (4) receiving influenza vaccines. In fact, the LPMWs with lower education in this study did adopt the latter three prevention measures less frequently. On the other hand, more highly educated participants believed that the outbreaks of AI in China would affect Taiwanese, and thus supported the ban on slaughtering live poultry in markets.

A ban on slaughtering live poultry in the market, first proposed in Hong Kong [40], has been implemented by the revised food commerce law [40]. In 2008, a woman in Beijing without prior contact with live birds was infected with AIVs after purchasing live poultry in a traditional market [12], implying that LPMs are one of the sources of AIV infection. Furthermore, most of the severe human H7N9 cases in 2013–2014 also acquired their infections through contact with poultry or visiting wet markets [10]. Although the Taiwan government initiated the implementation of the policy to ban slaughtering live poultry in LPMs on April 1st, 2008 (e.g., after the H5N2 outbreak in Kaohsiung with controversial answers on viral pathogenicity), this was postponed, then reinstated on May 17, 2013 due to the occurrence of the first imported H7N9 case in Taiwan. In this study, less-educated, high-risk groups had lower RAP. Therefore, enhancing surveillance of AIVs in avian hosts as well as humans in LPMs [26], timely epidemiologic data analyses and prompt risk communication with evidence-based data support, focusing on changing the minds of lower-educated, high-risk groups, will be very helpful to quickly control novel influenza viruses, thus minimizing the occurrence of potential pandemics.

PPMs are particularly useful for reducing the risk of acquiring or transmitting emerging respiratory infections before the availability of commercial vaccines [41]. Education is the most cost-effective approach to deliver the correct knowledge and ways to prevent AIV infections and future epidemics. Highly educated persons who had better access to the information on AI from television, newspapers and the internet in this study had higher risk perception of AI, similar to the findings in China [16] and Afghanistan [42]. Most importantly, strict compliance with personal protective equipment (PPE) requirements must be reinforced to manage the outbreaks of AI, regardless of the pathogenicity of the virus [43, 44]. Incomplete use of PPE was also associated with conjunctivitis and influenza-like illness after the 2006 outbreak of LPAI H7N3 in Norfolk, England [21]. Generally, compliance with most PPE requirements tends to be suboptimal for the highly exposed groups [45]. Similarly, Taiwanese LPM-workers generally feel that wearing PPE is uncomfortable, and they have not gotten used to it. The low risk perception accompanying such poor PPE usage needs to be guided with solid examples to finally achieve behavioral change. Our study subjects who had higher risk perception of AI and who were more aware about the government’s new policy had more motivation to use self-protection for preventing AIVs. Therefore, it is necessary to improve public awareness about the government’s prevention policy and also educate different target groups with various approaches, based on educational levels, job duties, and residential areas.

Identifying populations with low acceptance rates of influenza vaccine is important before developing and implementing vaccination programs, particularly as the acceptance of influenza vaccines has become quite low in recent years in many parts of the world [46, 47]. We found that market workers and the respondents living in Taiwan (except the HPAI H5N2 outbreak sites in southern Taiwan) did not believe that any influenza vaccine provided effective protection against AIVs. A significant drop was observed in the perception of the vaccine effectiveness in preventing “avian influenza” after the impact of HPAI compared to LPAI H5N2 outbreaks for both LPMWs (6.9 % vs 20.2 %) and CRs (7.9 % vs 19 %). In addition, respondents who did not believe in the external influence of outbreaks of AI in Mainland China, as well as those who paid no attention to domestic policy in Taiwan, and those who found no need to protect themselves against AIV infection did not trust the effectiveness of any influenza vaccine for humans or poultry. To solve this problem, risk communication on sources of the risk as well as scientific data supporting safety becomes very important. Additionally, easy access of high-risk populations to AI vaccines (similar to the established system for seasonal influenza vaccination of schoolchildren and the elderly in Taiwan, supported by a well-established public health infrastructure), a feasible plan on resources allocation, and the available AI vaccine inducing higher immunogenicity through better innate immunity [48, 49] all together, with systematic approaches, will reduce human infections of AIVs.

There are three major limitations of this study. First, there is a possibility of selection bias caused by the willingness of respondents to reply to the questions in the survey, even though we covered most of the live poultry markets affected by the outbreaks, and the LPMWs’ response rates were 93–98 % and 80 % after the outbreaks of LPAI H5N2 and HPAI H5N2, respectively. In addition, the 2nd-stage survey was conducted only in central Taiwan, where the scale of layer chickens was the largest. Second, our results may show reduced RAP because more study subjects of LPMs in the 1st-stage survey came from northern Taiwan, where large-scale wholesale broiler chickens and ducks coming from different parts of Taiwan are sold with better management and hygienic standards, whereas high densities of poultry farms are located in central and southern Taiwan. Third, the study subjects of the 1st and 2nd surveys were different and not comparable, and those results indicate only association rather than causation, because of the cross-sectional study design. The outbreaks of LPAI H5N2 were larger in scale, and occurred much more frequently and in more places than those of HPAI H5N2. To protect participants’ privacy, we did not collect personal identification data, and therefore could not follow up on the respondents in the initial survey. Future research should focus on the most effective methods and contents for risk communication in order to target different risk groups. Risk perception problems on LPAIVs need to be explored in relation to the scale, breeding style, types, and sanitation of poultry farms and different kinds of LPMs, particularly in areas with limited resources and expertise. In addition, behavioral research is worth doing to direct the best prevention and control policies, considering the acceptance of influenza vaccination and acceptable behavior change in high-risk groups versus the general public.

## Conclusions

Based on our findings, we sincerely recommend that health agencies enhance additional routine two-way risk communication with friendly interpersonal guidance for live-poultry market workers, poultry butchers and farmers, and related high-risk groups, particularly before outbreaks of AI. In addition, to minimize political concerns, fatal human cases after infection with AIV, including LPAIVs of H7N9 and H10N8 [47] in other countries can serve as solid examples for education, using easily understandable wordings and movies to demonstrate the danger of aerosol transmission of AIVs. Above all, the policy on banning the slaughter of live poultry at LPMs supported by incentives of tax reduction or free health care or certification to win customers’ trust, as well as active virological and serological surveillance with random sampling in poultry farms and markets, could be the most efficient way to reduce cross-species transmission. In conclusion, person-to-person risk communication to high-risk groups using more acceptable and attractive approaches and effective public policies on “one health” [50], and post-policy evaluation with international comparison will be helpful to promote global health.

## Additional files

Additional file 1:
**Appendix 1.**
Additional file 2:
**Appendix 2.**
Additional file 3:
**Appendix 3.**
Additional file 4:
**Appendix 4.**
