# Supplementary material for: Changing risk awareness and personal protection measures for low to high pathogenic avian influenza in live-poultry markets in Taiwan, 2007 to 2012
Source: BMC Infect Dis. 2015 Jun 24;15:241. doi: 10.1186/s12879-015-0987-8 (PMC4478710; doi:10.1186/s12879-015-0987-8)
Supplement: Additional file 1: — Appendix 1. [file 12879_2015_987_MOESM1_ESM.docx]

**Appendix 1 Questionnaires (one-page long).**

**Survey on Understanding of Avian Flu in Live-poultry Markets in Taiwan**

**(A): Questionnaire for Workers (Risk Group) in the Live-poultry Markets**

(Designed by Graduate Institute of Epidemiology and Preventive Medicine, College of Public Health, National Taiwan University)

Date: ___ Area: __________(City/County)

Name of Market: ___

Interviewer: ___

1. Your **age**: ___ **Gender**: ___

2. **Levels of Education**: (0) Elementary school (or below) (1) Junior high (2) Senior high/vocational school (3) University (or above)

3. Your **job duties** (may choose more than one):

(1) Butchery (2) Cleaning (3) Transportation (4) Administrative (5) Selling flowers (6) Selling dried products (7) Selling seafood (8) Selling vegetables (9) Selling uncooked poultry meat (10) Selling cooked poultry meat (11) Selling pork (12) Selling beef (13) Selling lamb (14) Cooking and selling internal organs (15) Others ___

4. Do you know that avian flu may cause serious diseases and even death?

(0) Yes (1) No [For Stage 2 only]

5. Have you taken any measures to protect yourself from avian influenza viruses?

(0) Nothing at all (8) Taking more information

(1) Vaccination against human flu (9) Moving to another place

(2) Vaccination against H5N1 (10) Emmigration

(3) Wearing a facemask (11) Getting a new job

(4) Washing hands frequently (12) Cooperating with the government

(5) Taking Chinese medicine (13) Following what people say on TV

(6) Taking nutritional wine in soup (14) Taking Tamiflu

(7) More physical exercise (15) Others ___

6. Which type of avian flu outbreak in China do you think has a greater influence on Taiwan?

(0) Neither of them (1) Only the bird type (2) Only the human type (3) Both

7. Are you aware of the fact that the proposal to ban the slaughter of live poultry in traditional markets starting April 2010 has been canceled at the end of March 2010? (may chose more than one)

(0) No (1) Yes (a) From newspaper (b) From TV (c) From web news (d) From other people (e) From friends or relatives

8. What attitude do you hold toward the following proposal of the government – No slaughter of live poultry in traditional markets, but only in automated slaughterhouses?

(0) I don’t care (1) Supportive, because ___ (2) Against, because ___

9. Do you think there will be cases of humans inflected with avian flu in Taiwan?

(0) Absolutely not (1) Probably not (2) Maybe (3) Certainly

10. Do you know the government’s new policy “Ten No’s and Five Needs”? For instance, DO NOT eat raw poultry meat, and DO NOT buy poultry meat of unknown source? (see Appendix 2)

(0) No (1) Somewhat (2) Clearly  (9) Do not understand

11. What preventive measures will you take against avian flu?

(0) None. No need. (1) Washing hands right after dealing with live fowls

(2) Wearing a facemask in the market (3) Both of the above (4) Change clothes and shoes after leaving the market and before going home (5) Others ___

12. What do you think is the effectiveness of seasonal flu vaccines against human flu and bird flu?

(0) No effect at all (1) Reduces the chance of human flu (2) Reduces the chance of bird flu (3) Reduces the chance of human flu and bird flu

13. What do you think about the case, in June 2012, of a child returning to Hong Kong from China, infected with H5N1 and being in critical condition? [For Stage 2 only]

(0) No idea (1) This has nothing to do with Taiwan (2) Government should pay attention to this (3) Both people and the government should pay attention to this (4) Others ___

14. Have you heard anything about the outbreaks of highly pathogenic avian influenza H5N2 in Fang-yuan and Chu-tang, Changhua (located in central Taiwan), both being highly infectious cases? [For Stage 2 only]

(0) No (1) Yes

15. How did you get to know these cases (Source of Information)? [For Stage 2 only] (1) From TV (2) Newspaper (3) Internet (4) Broadcast (5) Workers in the market (6) Friends or relatives (7) Cell phone (8) Others ___

16. Faced with many infectious diseases in Taiwan, which of the following makes you take notice and take personal preventive measures (e.g. wearing a facemask or washing hands)? [For Stage 2 only]

(1) General bird flu H5N2 (2) Highly infectious H5N2 (3) New flu H1N1 (4) Enterovirus (5) SARS (6) Tuberculosis (7) Others ___

17. Do you think the highly pathogenic H5N2 viruses can cross over to infect human beings? [For Stage 2 only]

(0) Bird flu cannot cross over to humans (1) Only highly pathogenic H5N1 can cross over to humans, not H5N2 (2) Only highly pathogenic H5N2 can, but not lowly pathogenic ones (3) All those viruses can do (9) No idea

18. If there were vaccines for bird flu, would you consider vaccination?

(0) No (1) Yes (a) If they’re free of charge (b) I can do this at my own expense (2) Yes, if the government sends people to my place and gives me a shot free of charge

19. What sincere suggestions would you offer to the government in the war against bird flu? ___

[**Thank you for your time!**]

**High Risk Workers:** butchers, raw chicken/duck sellers.

**Moderate Risk Workers:** Sellers of cooked chicken/duck, beef, pork, mutton, and/or other raw meat.

**Low Risk Workers**: Market cleaners, administrative officers, and those selling flowers, dry goods, vegetables and fruits.
