# Supplementary material for: Changing risk awareness and personal protection measures for low to high pathogenic avian influenza in live-poultry markets in Taiwan, 2007 to 2012
Source: BMC Infect Dis. 2015 Jun 24;15:241. doi: 10.1186/s12879-015-0987-8 (PMC4478710; doi:10.1186/s12879-015-0987-8)
Supplement: Additional file 2: — Appendix 2. [file 12879_2015_987_MOESM2_ESM.docx]

**Appendix 2 The new government’s policy on “Ten No’s, Five Needs” to prevent human infections of AIVs.**

**Ten (10) No’s:**

1. Do NOT get close to, touch, or feed migrating birds, pet birds or poultry.
2. Do NOT go to bird flu epidemic areas to visit an exhibition of birds. Do NOT carry birds into Taiwan without clearance or report.
3. Do NOT allow pet birds to live together with other domesticated animals (chickens, ducks, pigs, etc.).
4. Do NOT release your pet birds (e.g. pigeons).
5. Do NOT purchase poultry meat from an unknown source.
6. Do NOT touch, sell, buy, or eat birds which are ill.
7. Do NOT throw away birds that die from illnesses without proper measures.
8. Do NOT slaughter birds by yourself.
9. Do NOT eat raw poultry products (including eggs and related products).
10. Do NOT go to crowded places with poor ventilation.

**Five (5) Needs:**

1. You NEED to wash your hands frequently: Wash hands with soap after any contact with bird meat or excrement.
2. Those who need to have long-term contact with poultry (veterinarians, poultry workers, sellers, and workers involved in transporting and slaughtering poultry) NEED to be vaccinated with a human flu vaccine.
3. You NEED to eat things cooked: Bird flu virus cannot tolerate heat. A temperature of 56^o^C for 3 hours, 60^o^C for 30 minutes, or 100^o^C for 1 minute will kill avian influenza viruses. Always eat chicken meat and eggs cooked.
4. You NEED to have a balanced diet, with enough exercise, sleep and rest, and less pressure.
5. You NEED to monitor your own health. Take your temperature every day. Once you get a fever, please put on a facemask and immediately go to see a doctor.
