# Supplementary material for: Changing risk awareness and personal protection measures for low to high pathogenic avian influenza in live-poultry markets in Taiwan, 2007 to 2012
Source: BMC Infect Dis. 2015 Jun 24;15:241. doi: 10.1186/s12879-015-0987-8 (PMC4478710; doi:10.1186/s12879-015-0987-8)
Supplement: Additional file 3: — Appendix 3. [file 12879_2015_987_MOESM3_ESM.docx]

**Appendix 3 Statistical tests used in the five and six questions during the surveys of Stage I and II.**

| **Stage 1** | | | | |
| --- | --- | --- | --- | --- |
| **Category** | **Outcome variables** | **Explanatory variables** | **Cox & Snell R Square** | **Nagelkerke R Square** |
| **Risk awareness** | 1. Taiwan will be affected by the outbreaks of influenza in China (stage1-Q1) | age, job, gender, education, area, agree or oppose banning on birds from being slaughtered in traditional markets, staeg1-Q2, stage1-Q3, stage1-Q4, stage-Q5 | 0.0357 | 0.1076 |
| **Risk awareness** | 2. Taiwan residents will become infected with avian influenza (AI) (stage1-Q2) | age, job, gender, education, area, agree/oppose banning on birds from being slaughtered in traditional markets,stage1-Q1, stage1-Q3, stage1-Q4,stage-Q5 | 0.1475 | 0.1966 |
| **Risk attitude** | 3. Knowledge of new “Ten No’s, Five Needs” policy (stage1-Q3) | age, job, gender, education, area, agree/oppose banning on birds from being slaughtered in traditional markets,stage1-Q1, staeg1-Q2, stage1-Q4,stage-Q5 | 0.1028 | 0.1227 |
| **Risk protection** | 4. Willing to take self-protection measures against AI viral infection (stage1-Q4) | age, job, gender, education, area, agree/oppose banning on birds from being slaughtered in traditional markets, stage1-Q1, staeg1-Q2,stage1-Q3,stage-Q5 | 0.0844 | 0.1841 |
| **Risk attitude** | 5. The vaccine will provide effective protection against AI viral infection (stage1-Q5) | age, job, gender, education, area, agree/oppose banning on birds from being slaughtered in traditional markets, stage1-Q1, staeg1-Q2,stage1-Q3, stage1-Q4 | 0.145 | 0.2847 |
| **Stage 2** | | | | |
| **Risk awareness** | 1.Taiwan will be affected by the outbreaks of influenza in China (stage2-Q1) | age, gender, job, education, stage2-Q2, stage2-Q3, stage2-Q4, stage2-Q5, stage2-Q6 | 0.257 | 0.557 |
| **Risk awareness** | 2.Taiwan residents will become infected with AI viruses (stage2-Q2) | age, gender, job, education, stage2-Q1, stage2-Q3, stage2-Q4, stage2-Q5, stage2-Q6 | 0.107 | 0.161 |
| **Risk attitude** | 3. Knowledge of new “Ten No’s, Five Needs” policy (stage1-Q3) | age, gender, job, education, stage2-Q1, stage2-Q2, stage2-Q4, stage2-Q5, stage2-Q6 | 0.098 | 0.136 |
| **Risk protection** | 4. Willing to take self- protection measures against AIV infection (stage2-Q4) | age, gender, job, education, stage2-Q1, stage2-Q2, stage2-Q3, stage2-Q5, stage2-Q6 | 0.139 | 0.312 |
| **Risk protection** | 5. Willing to receive AI vaccination (stage 2-Q5) | age, gender, job, education, stage2-Q1, stage2-Q2, stage2-Q3, stage2-Q4, stage2-Q6 | 0.093 | 0.131 |
| **Risk attitude** | 6. Know AI may cause serious illness and even death (stage 2-Q6) | age, gender, job, education, stage2-Q1, stage2-Q2, stage2-Q3, stage2-Q4, stage2-Q5 | 0.102 | 0.228 |

**Cox & Snell R Square Test:** This is the original R square test in SAS before adjustment.

**Nagelkerke R Square Test:** This is the adjusted R square test in SAS labeled as “Max-rescaled RSquare”.

**Reference**: Ernest S. Shtatland, Sara Moore, Mary B. Barton. Why we need an R^2^ measure of fit (and not only one) IN PROC LOGISTIC AND PROC GENMOD. http://www2.sas.com/proceedings/sugi25/25/st/25p256.pdf
