# Supplementary material for: Changing risk awareness and personal protection measures for low to high pathogenic avian influenza in live-poultry markets in Taiwan, 2007 to 2012
Source: BMC Infect Dis. 2015 Jun 24;15:241. doi: 10.1186/s12879-015-0987-8 (PMC4478710; doi:10.1186/s12879-015-0987-8)
Supplement: Additional file 4: — Appendix 4. [file 12879_2015_987_MOESM4_ESM.docx]

**Appendix 4 The recoding rules to assign dependent binary variables (response scales) in this study**

1. Which type of avian flu outbreaks in China would have greater influence on Taiwan? **(Binary as follows)**

(0) Neither of them🡪0

(1) Only the bird type🡪1

(2) Only the human type🡪1

(3) Both🡪1

1. Do you think there would be human cases infected with avian flu viruses in Taiwan? **(Binary as follows)**

(0) Absolutely not 🡪0

(1) Probably not🡪0

(2) Maybe🡪1

(3) Certainly 🡪1

1. Do you know the government’s new policy “Ten No’s and Five Needs”? For instance, DO NOT eat raw poultry meat, and DO NOT buy poultry meat of unknown source? **(see Appendix 2) (Binary as follows)**

(0) No🡪0

(1) Somewhat🡪1

(2) Clearly🡪1

1. What preventive measures will you take against avian flu? **(Binary as follows)**

(0) None. No need. 🡪0

(1) Washing hands right after dealing with live fowls 🡪1

(2) Wearing a facemask in the market🡪1

(3) Both of the above🡪1

(4) Change clothes/shoes after leaving the market & before going home🡪1

(5) Others -->Not counted

1. What do you think is the effectiveness of seasonal flu vaccines against human flu and bird flu? **(Binary as follows)**

(0) No effect at all🡪0

(1) Reduces the chance of human flu🡪1

(2) Reduces the chance of bird flu🡪1

(3) Reduces the chance of human flu and bird flu🡪1

**f)** Do you know that avian flu may cause serious disease and even death?

[For **Stage 2 only,** after the outbreak of HPAI] **(Binary as follows)**

**(0) Yes🡪1**

(1) No🡪0

All the response scales for **Tables 2 and 4** were summarized in this **Appendix 4.**
